# Tirzepatide for Excess Weight in Adults With Type 1 Diabetes Using MiniMed 780G

**DOI:** 10.1111/dom.71002

**Published:** 2026-06-16

**Authors:** Michelantonio De Fano, Rachele Tortoriello, Stefania Rucco, Sofia Corio, Marcello Manco, Edoardo Guerrini, Francesca Porcellati

**Affiliations:** ^1^ Endocrine and Metabolic Sciences Section, Department of Medicine and Surgery University of Perugia Perugia Italy

**Keywords:** antiobesity drug, CSII, incretin therapy, type 1 diabetes

## 1 Introduction

A significant shift in the clinical phenotype of individuals with type 1 diabetes mellitus (T1D) has occurred in recent decades, with a marked increase in the prevalence of overweight and obesity [1]. This evolving clinical scenario, together with the rising overall prevalence of T1D [2], calls for broader therapeutic strategies to address the emerging metabolic needs of this population.

The introduction of incretin‐based therapies has transformed the management of obesity and has already reshaped treatment approaches in type 2 diabetes [3]. Owing to their multiple metabolic effects, these agents may represent valuable adjunct therapies in T1D. Although early studies with liraglutide raised concerns regarding hypoglycaemia and ketosis [4, 5], more recent evidence from real‐world [6, 7] and a randomised‐controlled trial (RCT) [8] suggests that semaglutide may provide clinically meaningful benefits with an acceptable safety profile in selected individuals with T1D.

Tirzepatide, the first dual agonist of the glucagon‐like peptide‐1 and glucose‐dependent insulinotropic polypeptide receptors, represents the most recent advancement in incretin‐based therapy and has shown remarkable efficacy in reducing body weight and improving glycaemic control [9]. However, evidence regarding its effectiveness and safety in real‐world T1D populations with excess weight remains limited, particularly among users of advanced insulin delivery systems. We report a retrospective case series evaluating the short‐term metabolic effects of adjunctive once‐weekly tirzepatide for excess weight in adults with T1D using the MiniMed 780G advanced HCL system.

## 2 Methods

### 2.1 Study Population and Study Design

This retrospective single‐centre case series included 10 adults (*n* = 10) with T1D and overweight (BMI ≥ 27 kg/m^2^) or obesity (BMI ≥ 30 kg/m^2^), followed at the Endocrine and Metabolic Science Section of the University Hospital of Perugia, in whom tirzepatide was initiated for weight reduction between April 2025 and October 2025. Baseline and 3‐month post‐treatment clinical data were collected, including demographics, anthropometrics, continuous glucose monitoring (CGM) metrics and insulin requirements. CGM and insulin delivery data were extracted from the Medtronic CareLink platform.

### 2.2 Outcome Measures

The main variables assessed were body weight and BMI. Secondary variables included CGM metrics—time in range (TIR, 70–180 mg/dL), time below range 1 (TBR1, 69–54 mg/dL), time below range 2 (TBR2, < 54 mg/dL), time above range 1 (TAR1, 181–250 mg/dL), time above range 2 (TAR2, > 250 mg/dL), glucose management indicator (GMI), coefficient of variation (CV) and Glycaemic Risk Index (GRI). Insulin requirements were also evaluated, including total daily dose (TDD), basal insulin, prandial boluses and automated correction boluses, as well as reported carbohydrate (CHO) intake.

### 2.3 Statistical Analysis

Normality was assessed using the Shapiro‐Wilk test. Continuous variables with normal distribution are presented as mean ± standard deviation (SD), whereas non‐normal variables are expressed as median and interquartile range (IQR). Pre‐ and post‐treatment comparisons used paired *t*‐tests for normally distributed variables and Wilcoxon signed‐rank tests for non‐normal variables. Statistical analyses were performed using R software, version 4.5.2 (R Foundation for Statistical Computing, Vienna, Austria). In all tests, a two‐sided *p*‐value < 0.05 was considered statistically significant.

### 2.4 Baseline Characteristics

The cohort included 10 subjects (six females), with a median age of 47.0 years (IQR 36.5, 52.0) and a mean diabetes duration of 23.2 ± 9.6 years. Median weight was 96.5 (IQR 80.0, 101.8) kg, with a mean body mass index (BMI) of 31.7 ± 3.3 kg/m^2^. Eight individuals met BMI criteria for obesity (seven class 1, one class 2), while two were classified as overweight. Two participants were receiving antihypertensive therapy (ACE inhibitors), and five were treated with cholesterol‐lowering medications (statins and ezetimibe); notably, of the two over weight participants, one was on antihypertensive therapy and the other on cholesterol‐lowering treatment, respectively. No macrovascular or microvascular complications, including diabetic retinopathy or maculopathy, were reported.

Mean duration of insulin pump therapy was 15.2 ± 8.1 years. All participants had completed structured diabetes education and were proficient in carbohydrate counting. Baseline CGM metrics indicated generally good glycaemic control.

### 2.5 Weight Parameters

Tirzepatide was initiated at 2.5 mg once weekly in all participants, with six uptitrated to 5 mg after 4 weeks based on physician judgement to optimise clinical benefit.

Median body weight changed by −11.3 kg (IQR −14.4 to −8.2), corresponding to a 12.2% reduction (IQR −16.7 to −10.0) (*p =* 0.002), while mean BMI changed by −4.1 ± 2.2 kg/m^2^, corresponding to a 13.0% ± 7.2% reduction (*p* < 0.001) (Table 1).

**TABLE 1:** Changes in metabolic and therapeutic parameters on tirzepatide over 3 months.

	T0	T3	*∆*	% *∆*	*p*
Weight (kg)^a^	96.5 [80.0, 101.8]	76.0 [69.8, 90.9]	−11.3 [−14.4, −8.2]	−12.2 [−16.7, −10.0]	**0.002** ^c^
BMI (kg/m^2^)^b^	31.7 ± 3.3	27.6 ± 4.0	−4.1 ± 2.2	−13.0 ± 7.2	**< 0.001** ^d^
TIR 70–180 mg/dL (%)^b^	70.1 ± 12.1	85.9 ± 9.0	15.8 ± 9.0	24.5 ± 16.4	**< 0.001** ^d^
TBR1 69–54 mg/dL (%)^b^	1.8 ± 1.6	0.8 ± 1.3	−1.0 ± 1.2	NA^e^	**0.02** ^c^
TBR2 < 54 mg/dL (%)^a^	0 [0, 0]	0 [0, 0]	0 [0, 0]	NA^e^	1
TAR1 181–250 mg/dL (%)^b^	22.0 ± 8.4	11.6 ± 7.2	−10.4 ± 6.9	−48.9 ± 27.2	**0.001** ^c^
TAR2 > 250 mg/dL (%)^b^	6.0 ± 5.2	1.7 ± 1.8	−4.3 ± 3.8	−73.2 ± 24.1	**0.006** ^c^
CV (%)^b^	30.9 ± 3.3	26.2 ± 4.1	−4.7 ± 2.0	−15.6 ± 7.1	**< 0.001** ^d^
GMI (%)^b^	6.9 ± 0.5	6.6 ± 0.3	−0.3 ± 0.4	−4.1 ± 5.1	**0.04** ^c^
GRI^b^	31.8 ± 12.7	13.9 ± 9.2	−17.9 ± 10.0	−58.2 ± 22.9	**< 0.001** ^d^
TDD (U/day)^b^	59.8 ± 31.4	33.9 ± 18.9	−25.9 ± 23.2	−40.1 ± 18.1	**0.006** ^c^
Basal dose (U/day)^b^	28.4 ± 14.2	17.9 ± 10.0	−10.5 ± 9.7	−32.7 ± 23.8	**0.01** ^c^
Prandial dose (U/day)^b^	31.3 ± 18.8	15.9 ± 9.6	−15.4 ± 14.7	−45.8 ± 15.9	**0.01** ^c^
Automatic correction dose (U/day)^b^	12.4 ± 9.7	5.1 ± 4.5	−7.3 ± 8.4	−53.1 ± 24.7	**0.02** ^c^
Reported CHO intake (g/day)^b^	164.1 ± 53.7	117.5 ± 47.6	−46.6 ± 50.3	−23.7 ± 25.7	**0.02** ^c^

*Note:* Data are presented as mean ± SD or median [Q1, Q3]. Bold values indicate statistically significant differences.

Abbreviations: % *∆*, delta in percentage; *∆*, delta; BMI, body mass index; CHO, carbohydrates; CV, coefficient of variation; g, grams; GMI, glucose management indicator; GRI, Glycaemia Risk Index; NA, not applicable; TAR, time above range; TBR, time below range; TDD, total daily dose; TIR, time in range; U, units.

^a^ Wilcoxon signed‐rank tests.

^b^ Paired *t*‐tests.

^c^ *p*‐value < 0.05.

^d^ *p*‐value < 0.001.

^e^ The percentage change was not calculable for parameters with 0% baseline values.

At follow‐up, only two individuals remained in the obesity range, whereas three reached normal weight, including one with baseline BMI > 30 kg/m^2^. Overall, nine individuals achieved ≥ 5% weight loss and seven achieved a reduction of at least 10% (Table S1).

### 2.6 Glycaemic Outcomes

Over 3 months, tirzepatide treatment was significantly associated with increased TIR (70.1% ± 12.1% to 85.9% ± 9.0%, *p* < 0.001), decreased TBR1 (1.8% ± 1.6% to 0.8% ± 1.3%, *p* = 0.02), decreased TAR1 (22.0% ± 8.4% to 11.6% ± 7.2%, *p* = 0.001) and decreased TAR2 (6.0% ± 5.2% to 1.7% ± 1.8%, *p* = 0.006) (Table 1; Figure S1). No differences were found for TBR2.

Concurrently, GMI decreased significantly (6.9% ± 0.5% to 6.6% ± 0.3%, *p* = 0.04), as well as CV (30.9% ± 3.3% to 26.2% ± 4.1%, *p* < 0.001) and GRI (31.8 ± 12.7 to 13.9 ± 9.2, *p* < 0.001) (Table 1).

### 2.7 Insulin Requirements and Carbohydrate Intake

**FIGURE 1:**
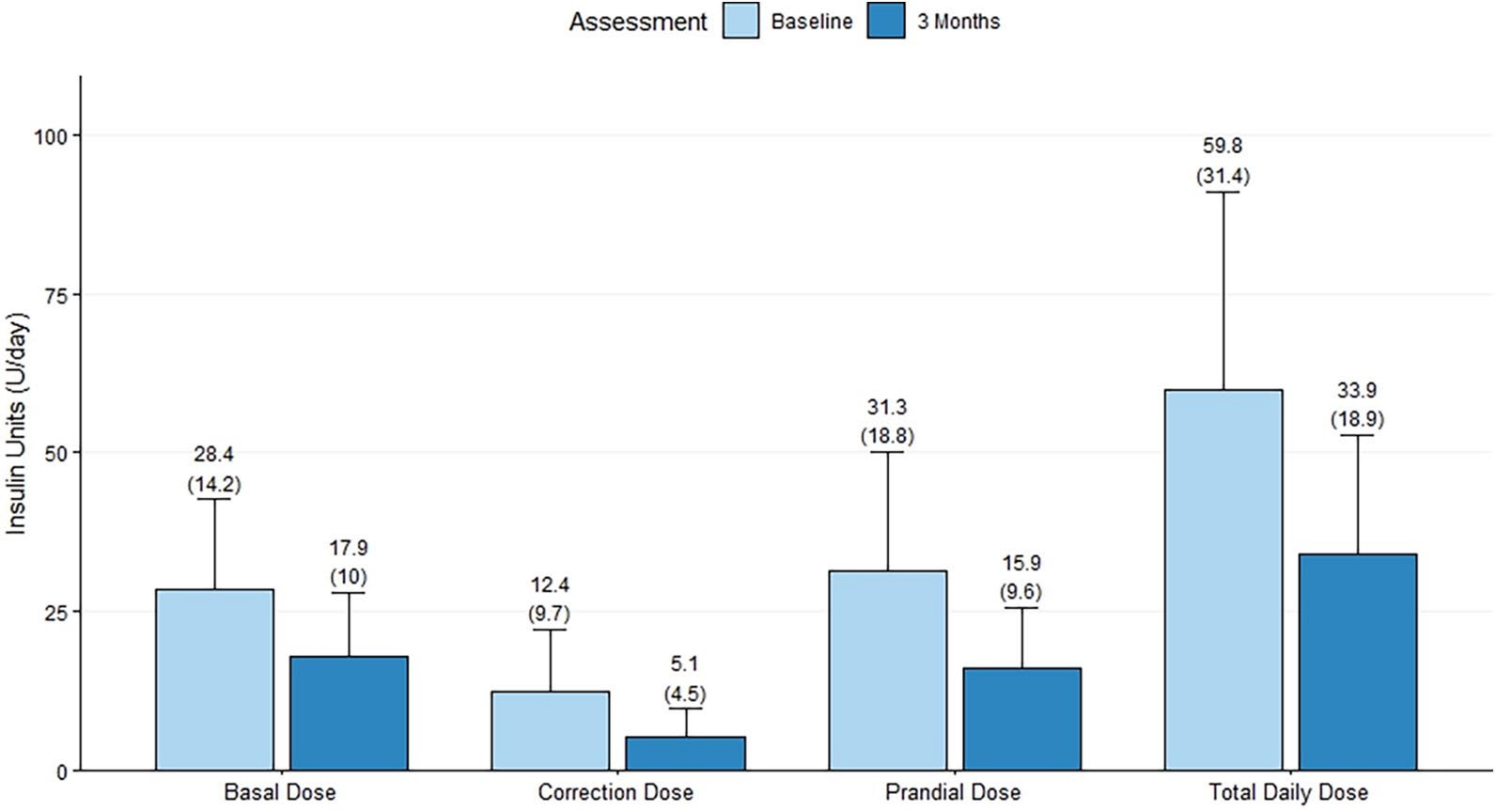
Changes in all components of insulin requirements over 3 months of tirzepatide treatment. Data are expressed as mean (SD). U = Units.

A significant reduction in TDD was observed (59.8 ± 31.4 to 33.9 ± 18.9 units/day, *p* = 0.006). Consistent reductions were observed across all insulin components: basal insulin (28.4 ± 14.2 to 17.9 ± 10.0 units/day, *p =* 0.01), prandial insulin (31.3 ± 18.8 to 15.9 ± 9.6 units/day, *p* = 0.01) and automatic correction boluses (12.4 ± 9.7 to 5.1 ± 4.5 units/day, *p* = 0.02) (Table 1; Figure 1). Notably, these changes occurred concurrently with a significant decrease in reported CHO intake, which dropped from 164.1 ± 63.7 to 117.5 ± 47.6 g/day (*p* = 0.02) (Table 1).

### 2.8 Safety

No participants discontinued tirzepatide due to gastrointestinal symptoms, and no other adverse events were reported. Furthermore, no hospitalisations or emergency visits occurred for hyperglycaemia, hypoglycaemia or diabetic ketoacidosis (DKA).

## 3 Discussion

This retrospective case series suggests that adjunctive tirzepatide in adults with T1D and excess weight using the MiniMed 780G system is associated with early improvements in weight control. Concurrently, tirzepatide induced significant improvements in CGM metrics, despite the cohort already showing good baseline glycaemic control.

Our real‐world findings are consistent with previous reports [10, 11], with the added value of focusing exclusively on individuals using MiniMed 780G and providing data on the GRI, a relatively novel and comprehensive CGM‐derived metric [12].

A similar pattern was observed for insulin requirements, which are often implicated in weight gain in this population. TDD decreased markedly within 3 months, with significant reductions in both basal and prandial boluses. Notably, automatic correction boluses, an essential component of MiniMed 780G functioning, also showed a statistically significant reduction.

These metabolic and therapeutic changes occurred alongside a significant decrease in CHO intake. This might reflect reduced hunger secondary to the direct effects of tirzepatide treatment, but potentially also a decreased need for carbohydrate ingestion to treat hypoglycaemia, although the latter was already infrequent at baseline in our cohort.

A recent 12‐week Phase 2 randomised placebo‐controlled trial (TIRTLE1) evaluated tirzepatide in adults with T1D, using the same doses as in our cohort. The trial demonstrated reductions in body weight (primary outcome) and improvements in glycaemia [13]. Among 12 participants receiving tirzepatide, six used insulin pumps, including three using the MiniMed 780G; exploratory analysis in these pump users showed that insulin dose decreased as early as 2 weeks into tirzepatide treatment, which was associated with body weight reduction. Regarding safety, assessed at every clinical visit during the individualised follow‐up, the absence of increased hypoglycaemia and DKA episodes despite insulin reduction provides reassuring signals for the use of tirzepatide in T1D.

Study limitations include the retrospective, single‐centre design and the small sample size, which may introduce selection bias. The lack of a control group precludes causal inference, while missing HbA1c and waist circumference data represent additional limitations. Furthermore, our results aggregate individuals on both 2.5 and 5 mg doses without dose‐dependent stratification or a mandatory dose escalation algorithm, as is standard in RCTs. This approach is a direct consequence of the real‐world setting, where dose titration is guided by a pragmatic approach of clinical judgement based on individual response and tolerability. While this reflects routine practice, it constitutes a significant methodological limitation. Consequently, these 3‐month results capture only the initial titration phase; while individualised weight stabilisation remains a primary long‐term objective, it was not a pre‐defined target for this short‐term observation, which focused instead on the early metabolic benefits and the clinical feasibility of the therapeutic approach.

Notably, even in our small cohort, clinical responses varied significantly: as illustrated in Table S1, one individual lost more than 25% of baseline weight while another lost less than 3%, despite the same dose titration. It would be of great interest to perform a detailed analysis of the determinants of weight loss for each participant, especially in light of the clinical concerns raised in recent years [14]. However, body composition assessment was not performed, as it is not yet completely part of the routine metabolic assessment in our centre. This represents another key limitation, alongside the lack of additional uniform follow‐up evaluations, such as fundus oculi examinations, although no participants had retinopathy at baseline.

Further information on these aspects will be provided by ongoing and future prospective studies, ideally featuring longer follow‐up periods. Such research should also evaluate whether these findings are consistent across different HCL users and when compared with individuals not using automated insulin delivery systems.

## 4 Conclusions

In the context of rising obesity among individuals with T1D, our observations provide encouraging evidence that tirzepatide is associated with meaningful metabolic benefits in users of MiniMed 780G. Broader clinical adoption will require longer‐term data and clear guidelines, but these findings highlight the potential to expand treatment options in a timely and clinically relevant manner.

## Author Contributions

All authors contributed to data collection. M.D.F. performed statistical analysis. M.D.F. and R.T. drafted the initial version of the manuscript. All authors critically revised the manuscript and approved the final version for submission.

## Funding

The authors have nothing to report.

## Conflicts of Interest

M.D.F. has received honoraria for lecturing and travel grants from AstraZeneca, Eli‐Lilly, Novo Nordisk, Sanofi. R.T. has received honoraria for travel grants from Eli‐Lilly, Novo Nordisk, Laboratori Guidotti. S.R. has received honoraria for travel grants from AstraZeneca, Novo Nordisk, Abbott. S.C. has received honoraria for travel grants from Eli‐Lilly and Novo Nordisk. M.M. has received honoraria for travel grants from Novo Nordisk and Laboratori Guidotti. E.G. has received honoraria for travel grants from Novo Nordik and Laboratori Guidotti. F.P. receives clinical trial funding, advisory board and lecture fees from Abbott, AstraZeneca, Eli‐Lilly, Novo Nordisk, Roche, Sanofi.

## Supporting information


**Table S1:** Individuals change in body weight, BMI and tirzepatide dose after 3 months.
**Figure S1:** Overall changes in CGM‐derived metrics following 3 months of tirzepatide treatment.

## Data Availability

Data available on request due to privacy/ethical restrictions.
