# Supplementary material for: Tirzepatide for Excess Weight in Adults With Type 1 Diabetes Using MiniMed 780G
Source: Diabetes Obes Metab. 2026 Jun 16;28(9):8709–13. doi: 10.1111/dom.71002 (PMC13448885; doi:10.1111/dom.71002)
Supplement: Supplementary file 1 [file DOM-28-8709-s001.docx]

**Supplementary**

**TABLE S1.** Individuals change in body weight, BMI, and tirzepatide dose after 3 months.

| Subject | Age (years) | Sex | Years of T1D | Weight (kg)  Baseline | BMI (kg/m^2^)  Baseline | Weight (kg)  3 months | BMI (kg/m^2^)  3 months | TZP dose at 3 months |
| --- | --- | --- | --- | --- | --- | --- | --- | --- |
| 1 | 30 | F | 22 | 101.0 | 38.5 | 90.5 | 34.5 | 5 |
| 2 | 52 | F | 12 | 76.0 | 31.2 | 68.5 | 28.1 | 5 |
| 3 | 53 | F | 18 | 78.0 | 31.2 | 73.4 | 29.4 | 5 |
| 4 | 34 | F | 8 | 80.0 | 27.7 | 68.6 | 23.7 | 2.5 |
| 5 | 44 | M | 22 | 102.0 | 31.5 | 75.2 | 22.7 | 5 |
| 6 | 32 | F | 25 | 93.5 | 34.3 | 76.8 | 28.2 | 2.5 |
| 7 | 52 | M | 35 | 104.4 | 31.5 | 93.3 | 28.2 | 2.5 |
| 8 | 53 | M | 38 | 105.5 | 30.5 | 91.0 | 26.3 | 5 |
| 9 | 47 | F | 32 | 80.0 | 27.0 | 66.0 | 22.3 | 2.5 |
| 10 | 47 | M | 20 | 99.5 | 33.5 | 97.0 | 32.8 | 5 |

Abbreviations: T1D = Type 1 diabetes; BMI = Body Mass Index; TZP = Tirzepatide.

**FIGURE S1.** Overall changes in CGM-derived metrics following 3 months of tirzepatide treatment.

Abbreviations: CGM = Continuous Glucose Monitoring; TIR = Time In Range 70-180 mg/dL; TBR1 = Time Below Range 69-54 mg/dL; TAR1= Time Above Range 181-250 mg/dL; TAR2 = Time Above Range >250 mg/dL.
